# Immunotherapy against the Cystine/Glutamate Antiporter xCT Improves the Efficacy of APR-246 in Preclinical Breast Cancer Models

**DOI:** 10.3390/biomedicines10112843

**Published:** 2022-11-08

**Authors:** Giuseppina Barutello, Antonino Di Lorenzo, Alessandro Gasparetto, Chiara Galiazzi, Elisabetta Bolli, Laura Conti, Federica Cavallo

**Affiliations:** Molecular Biotechnology Center “Guido Tarone”, Department of Molecular Biotechnology and Health Sciences, University of Turin, Via Nizza 52, 10126 Turin, Italy

**Keywords:** xCT, APR-246, breast cancer, p53, cancer immunotherapy

## Abstract

Breast cancer is the most frequent cancer in women. Despite recent clinical advances, new therapeutic approaches are still required. The cystine-glutamate antiporter xCT, encoded by the *SLC7A11* gene, which imports cystine in exchange with glutamate, is a potentially new target for breast cancer therapy, being involved in tumor cell redox balance and resistance to therapies. xCT expression is regulated by the oncosuppressor p53, which is mutated in many breast cancers. Indeed, mutant p53 (mut-p53) can induce xCT post-transcriptional down modulation, rendering mut-p53 tumors susceptible to oxidative damage. Interestingly, the drug APR-246, developed to restore the wild-type function of p53 in tumors harboring its mutation, alters the cell redox balance in a p53-independent way, possibly rendering the cells more sensitive to xCT inhibition. Here, we propose a combinatorial treatment based on xCT immunetargeting and APR-246 treatment as a strategy for tackling breast cancer. We demonstrate that combining the inhibition of xCT with the APR-246 drug significantly decreased breast cancer cell viability in vitro and induced apoptosis and affected cancer stem cells’ self-renewal compared to the single treatments. Moreover, the immunetargeting of xCT through DNA vaccination in combination with APR-246 treatment synergistically hinders tumor progression and prevents lung metastasis formation in vivo. These effects can be mediated by the production of anti-xCT antibodies that are able to induce the antibody dependent cellular cytotoxicity of tumor cells. Overall, we demonstrate that DNA vaccination against xCT can synergize with APR-246 treatment and enhance its therapeutic effect. Thus, APR-246 treatment in combination with xCT immunetargeting may open new perspectives in the management of breast cancer.

## 1. Introduction

Breast cancer has recently become the most frequently diagnosed cancer worldwide, with more than 2.2 million new cases in 2020. Although the introduction of new treatment options has improved the outcome of breast cancer patients, this disease remains the first cause of cancer deaths in women globally [1]. Indeed, cancer metastases and resistance to therapy still develop in many patients and represent important hindrances to the successful treatment of breast cancer [2]. Therefore, new target molecules and new strategies are needed for patients with aggressive and resistant forms of breast cancer [3]. We have previously identified xCT, the light chain of the antiporter system xc-, which imports cystine in exchange with glutamate, as a potential new target for breast cancer therapy [4]. xCT is a multipass transmembrane protein encoded by the *SLC7A11* gene, expressed at low levels in healthy tissues but highly expressed in several tumors, including all breast cancer subtypes [4,5]. xCT plays a key role in breast cancer progression, particularly in metastatic dissemination, since the exported glutamate, by activating the metabotropic glutamate receptor 3, induces the Rab27-mediated expression of the matrix metalloproteinase MT1-MMP on the cell surface, which degrades the extracellular matrix, favoring invasion [6]. xCT also protects cancer cells from the oxidative stress, rendering them resistant to most current chemotherapies. Inside the cell, cystine is reduced to cysteine, which is necessary for the synthesis of glutathione (GSH), a very important antioxidant molecule. By reducing intracellular reactive oxygen species (ROS), GSH confers chemoresistance to cancer cells and prevents their ferroptosis, senescence, autophagy, and differentiation [5]. Hence, xCT targeting may potentiate breast cancer therapies.

Some pharmacological inhibitors of xCT have been developed, including Erastin [7], Erastin Ketone Imidazole (IKE) [8], and sulfasalazine (SAS) [9]. SAS has been approved by the Food and Drug Administration (FDA) for the treatment of several inflammatory diseases [7]. However, these drugs are not specific for xCT when administered in vivo and have poor solubility and pharmacokinetics, leading to many unwanted effects [5,7,10]. To overcome these limitations, we have previously developed several vaccines against xCT based on plasmid DNA, viral vectors, or virus-like particles that induce an immune response able to attack cancer cells. These vaccines significantly counteracted the metastatic dissemination in preclinical models of breast cancer, while the effect on primary tumor growth was less striking [4,11,12,13]. This observation suggested that xCT targeting might be more useful as part of a combinatory treatment than as a single agent, spurring us to identify those therapies that would benefit from an association with anti-xCT vaccination. We thus demonstrated that xCT vaccination potentiates the efficacy of HER2-targeted therapies in preclinical models of mammary cancer [14]. Additional combined therapies should be developed for the other breast cancer subtypes.

xCT is a central player in the crosstalk between redox balance, cell metabolism, and oncogenetic mechanisms [15]. A link between xCT and the oncosuppressor p53 has been demonstrated. xCT can be downregulated by wild type p53, which binds to a consensus sequence located 5′ to the transcription start site of the *SLC7A11* gene [16] and by inducing the deubiquitination of histone H2B at lysine 120 (H2Bub1), which represses *SLC7A11* transcription [17]. Moreover, gain of function missense mutations of the *TP53* gene (mut-p53), observed in about 30% of all breast cancer cases and in 80% of triple negative breast cancers (TNBC) [18], induce the downregulation of xCT by impairing the function of the nuclear factor erythroid 2-related factor (NRF2), the main activator of xCT transcription [19]. Besides regulating the cell redox balance in this way, mut-p53 promotes cancer progression by inducing proliferation and increasing stemness, epithelial to mesenchymal transition (EMT), invasion, and migration [18]. Therefore, several strategies to restore the p53 function in cells harboring mut-p53 have been developed, such as drugs that are able to restore wt-p53 conformation and function. Among these, APR-246 (also known as methylated p53-reactivation and the induction of massive apoptosis-1, PRIMA-1^met^, or Eprenetapopt) is currently undergoing clinical trials in patients with several tumors [20,21,22]. Of note, the p53 mutational status is not the only predictive condition for the therapeutic response to APR-246 [23]. Indeed, besides reactivating wt-p53 functions, APR-246 induces oxidative stress in the cells independently from their *TP53* status. Inside the cell, APR-246 is rapidly converted into its active compound methylene quinuclidinone (MQ), which covalently binds GSH and blocks its activity [24]. Thus, although the mutant status of p53 connotes with increased oxidative stress due to a decreased xCT expression, restoration of p53 activity with APR-246 may further aggravate the cell oxidative stress by inactivating GSH. On the other hand, however, treatment with APR-246 may induce xCT expression both directly, by impairing the mut-p53-dependent NRF2 inhibition of xCT, and indirectly, by increasing ROS and consequently inducing the upregulation of the genes involved in the redox balance. xCT upregulation, by restoring the redox balance, may antagonize the therapeutic activity of APR-246 [25,26]. Thus, the pharmacologic inhibition of xCT using SAS, further impairing the cellular redox balance, may exert a synergistic anticancer effect when applied in combination with APR-246 administration [25]. Indeed, it has been demonstrated that the expression level of xCT inversely correlates with the sensitivity to APR-246, suggesting xCT to be a useful prognostic marker for APR-246 treatment response [23,25].

Based on these data, we hypothesized that APR-246 treatment might improve the efficacy of xCT targeting. Therefore, we tested the effects of combining SAS and APR-246 for the treatment of breast cancer cell lines harboring different *TP53* states in vitro, demonstrating that their combination was more effective than the single treatments in reducing cancer cell viability, inducing apoptosis, and affecting cancer stem cell (CSC) self-renewal. Moreover, the combination of a DNA-based vaccine targeting xCT with APR-246 administration synergistically hindered tumor progression and inhibited lung metastasis formation in preclinical models of breast cancer independently from the presence of mut-p53. These effects were mediated by the production of antibodies targeting xCT that are able to induce Antibody Dependent Cellular Cytotoxicity (ADCC). These results may offer new perspectives into the management of breast cancer.

## 2. Materials and Methods

### 2.1. Cell Cultures

Four breast carcinoma cell lines harboring different p53 mutational states were used: 4T1 (triple negative; p53 null) [27] and TS/A (Her2+; p53^R270H^) [28] mouse mammary cancer cell lines; MCF-7 (ER+; p53 wild-type) and MDA-MB-231 (triple negative; p53^R280K^) human breast cancer cell lines [29]. MDA-MB-231, MCF-7, and 4T1 were purchased from American Type Culture Collection (ATCC; Manassas, VA, USA) in 2018, aliquoted, frozen, and then used within 10 passages after resuscitation in Dulbecco’s modified eagle medium (DMEM) or RPMI 1640 (ThermoFisher Scientific, Waltham, MA, USA) 10% FBS (Sigma–Aldrich , St. Louis, MO, USA), respectively. TS/A are cell lines derived from an HER2+ mammary cancer spontaneously arisen in BALB/c mice [30]; they were cultured in RPMI 10% FBS. All cells tested negative for mycoplasma. Tumorspheres were generated and maintained as in [31] and cultured in serum-free DMEM-F12 medium (ThermoFisher Scientific) supplemented with 20 ng/mL basic fibroblast growth factor (bFGF), 20 ng/mL epidermal growth factor (EGF), 5 µg/mL insulin, and 0.4% bovine serum albumin (all from Sigma-Aldrich).

### 2.2. Cell Viability Assay

Cells (7 × 10^3^) were left overnight in complete medium in 96-well plates. Scalar or fixed doses of APR-246 (Syngene; Bangalore, India) or Erastin (0.2 μM; APExBIO; Boston, MA, USA), alone or in combination with 100 μM SAS, were then added, and cells were incubated for 24 h. 3-(4,5-dimethylthiazol-2-yl)-2,5-diphenyltetrazolium bromide (MTT; 0.5 mg/mL) was added for 4  h at 37 °C, then the supernatant was removed and 150 µL of dimethyl sulfoxide (Sigma-Aldrich) was added to dissolve formazan crystals. Absorbance was measured on a 680XR microplate reader (Bio-Rad, Hercules, CA, USA) at 570 nm and 650 nm (background subtraction). 4T1 and TS/A cells, plated in 96-well plates in antibiotic-free medium (7 × 10^3^ density), left to adhere, and then transfected with 200 nM of a pool of three stealth siRNA sequences used to silence *SLC7A11* (MSS218649, MSS218650, MSS281945), or the corresponding negative control (scrambled), were purchased from ThermoFisher. After 24 h, cells were treated or not with APR-246. Cell viability was assessed by MTT assay 24 h later, as described above.

### 2.3. Immunoblot

As previously described [32], total protein cell lysates of cells treated or not with APR-246 for 16 h were obtained using a cold RIPA buffer (150 mM NaCl; 50 mM Tris-HCl pH 8.00; Sodium dodecyl sulphate 0.1%; Sodium Deoxycholate, 0.5%; Nonidet P-40 1%) mixed with protease and phosphatase inhibitors (Sigma-Aldrich). Following 30 min incubation at room temperature in 2-Mercaptoethanol-containing Laemmli Sample Buffer (Bio-Rad) plus 2 min denaturation at 95 °C, equal amounts of samples were loaded onto polyacrylamide-precast gel (Bio-Rad) and transferred to PVDF membranes (Millipore, Burlington, MA, USA). Membranes were incubated in blocking buffer (5% non-fatted milk or 5% Bovine Serum Albumin in TBS-T 0.1%; from Santa Cruz Biotechnology, Santa Cruz, CA, USA, and Sigma-Aldrich, respectively) and probed overnight at 4 °C with antibodies to mouse xCT, human xCT, or vinculin (all from Cell Signaling Technology, Danvers, MA, USA) and used as loading control. After washing, horse radish peroxidase (HRP)-conjugated secondary antibodies (Sigma-Aldrich) were used. The chemiluminescent signal was detected using ECL (Cyanagen, Bologna, Italy) through the ChemiDoc™ Touch Imaging System (Bio-Rad).

### 2.4. Cancer Stem Cell Self-Renewal Assay

4T1, TS/A, MCF7, and MDA-MB-231 first passage tumorspheres were disaggregated, and the resulting cells were plated in tumorsphere growth medium at a concentration of 5 × 10^4^ cells/mL in a Corning^®^ Ultra-Low attachment 96-well plate (Thermo Fisher Scientific) and treated with APR-246 (5 µM), SAS (150 µM), or a combination of the two drugs. The number of tumorspheres (round-shaped cell aggregates presenting a diameter >80 µm) per well were counted after 6 days through a Leica (Teaneck, NJ, USA) DMi1 inverted microscope (4× magnification) and summed; then, the total number of tumorspheres was calculated and the number of tumorspheres generated per 1 × 10^4^ cells seeded was calculated. Data were expressed as the percentage of the reduction in the tumorspheres’ forming ability compared to the cells cultured in medium [31].

### 2.5. FACS Analysis

Cells were plated (3 × 10^4^ cells/well) in a 24-well plate and left to adhere for 24 h before being treated with APR-246 5 μM and SAS 100 μM, alone or in combination, for a further 24 h. Cells were then collected, and intracellular reactive oxygen species (ROS) were stained with 2′,7′-dihydrochlorofluorescein diacetate (Sigma-Aldrich, cod. 35848) [4]. Apoptosis was evaluated with AnnexinV-Apoptosis Kit-APC (eBioscience, San Diego, CA, USA; cod. 88-8007-72) following the manufacturer’s instructions. Briefly, cells were treated with APR-246 5 μM and/or SAS 100 μM for 24 h, collected, washed with Ca2^+^-containing binding buffer, and incubated for 10 min at room temperature with Annexin V-APC. After washing, PI was added, and the cells were analyzed by fluorescence-based flow cytometry (FACS). Annexin V+ PI- cells were considered apoptotic [33]. To evaluate xCT, the cells were fixed/permeabilized with a BD Cytofix/Cytoperm kit (BDBioscience, Milano, Italy) and stained with anti-xCT rabbit antibody (PA1-16775, Thermo Fisher Scientific) followed by FITC-anti-rabbit-Ig (Dako, Jena, Germany) [14]. Cells were acquired on a BD-FACSVerse (BDBioscience) and analyzed with FlowJO version 10.5.3 software (FlowJo, Ashland, OR, USA).

### 2.6. In Vivo Treatments

BALB/c mice were bred and maintained under saprophytic and pathogen-free conditions at the animal facility of the Molecular Biotechnology Center (University of Turin) and treated in accordance with EU and institutional guidelines, with the approval of the Animal Care and Use Committee of University of Turin and Italian Ministry of Health (authorization 500/2017-PR).

Tumors were induced by injecting 1 × 10^4^ 4T1 or TS/A cells in the corresponding fourth mammary gland in 8-week old BALB/c female mice. Tumor growth was monitored twice per week using a caliper. When the tumors reached 2 mm mean diameter, the mice were blindly randomized into four groups, which were vaccinated with a mouse xCT coding plasmid (mxCT) or the empty pVAX plasmid as a control. Vaccination consisted of an intramuscular injection of 50 μg of plasmid, followed by electroporation as described in [4]. Vaccination was repeated a week after. Starting from the first vaccination and until the end of the experiment, mice were treated daily and intraperitoneally with 100 mg/Kg APR-246, or with the vehicle as a control. Tumor growth was monitored once per week with a caliper and was reported as tumor volume, calculated as volume of sphere (V = 4/3 πr³). When the tumors of the control mice reached 10 mm mean diameter, mice were culled and their lungs were fixed in formalin before being paraffin embedded and stained with H&E. Micrometastases were counted on a Nikon SMZ1000 stereomicroscope (Mager Scientific, Dexter, MI, USA), and representative images acquired on an Olympus BX41 microscope (Olympus, Breinigsville, PA, USA) at 2.5× magnification [4].

### 2.7. ELISA Assay

Serum was collected one week after the second vaccination by retro-orbital bleeding. Sera (1:50 dilution) were incubated on microplates previously coated with recombinant mouse xCT protein (Cloud-Clone-Corp., Katy, TX, USA; 40 ng/well) and binding was detected with HRP-conjugated-anti-mouse-IgG antibody (Sigma-Aldrich) using a 680XR microplate reader (Bio-Rad), as previously described [34].

### 2.8. Antibody Dependent Cell Cytotoxicity (ADCC)

1 × 10^4^ 4T1 or TS/A target cells stained with 2 µM of the fluorescent cell staining dye carboxyfluorescein succinimidyl ester (CFSE, Molecular Probes, Eugene, OR, USA) were cultured with splenocytes (SPC) from syngeneic BALB/c mice at effector:target (E:T) ratios of 200:1, 100:1, and 50:1 overnight in the presence of sera from vaccinated mice (1:50 dilution). Cells were then detached, stained with 1 μg/mL 7-Amino-ActinomycinD (7-AAD, BDBiosciences), and analyzed by FACS. The percentage of ADCC was calculated as in [12] and reported and compared to that produced by the sera from mice vaccinated with pVAX alone.

### 2.9. Statistical Analysis

Statistical significance was evaluated using GraphPad9 software (GraphPad Software, Inc., San Diego, CA, USA). Differences in data from sphere formation, cell viability, FACS, an enzyme-linked immunosorbent assay (ELISA) test, and tumor volume, considered at the experimental endpoint, were analyzed using two-tailed unpaired Student’s *t* test (with Welch’s correction for samples with different variance) and nonparametric Mann-Whitney or Kruskal-Wallis test when the distribution calculated using the Shapiro-Wilk or Kolmogorov Smirnoff tests was not normal. *p* < 0.05 was considered significant.

## 3. Results

### 3.1. Pharmacological Inhibition of xCT Combined with APR-246 Treatment Effectively Reduces Tumor Cell Viability by Altering Intracellular Redox Balance

To point out the effect of the dual treatment encompassing the inhibition of xCT activity and the reactivation of mut-p53, we employed different breast carcinoma cell lines harboring different p53 mutational states and expressing xCT, as determined by cytofluorimetric analyses (Supplementary Figure S1): 4T1 (triple negative; p53 null) [27] and TS/A (Her2+; p53^R270H^) [28] mouse mammary cancer cell lines; MCF-7 (ER+; p53 wild-type) and MDA-MB-231 (triple negative; p53^R280K^) human breast cancer cell lines [29]. Cells were cultured in the presence of scalar doses of APR-246, alone or in combination with SAS, and then cell viability was measured in an MTT assay.

**Figure 1:**
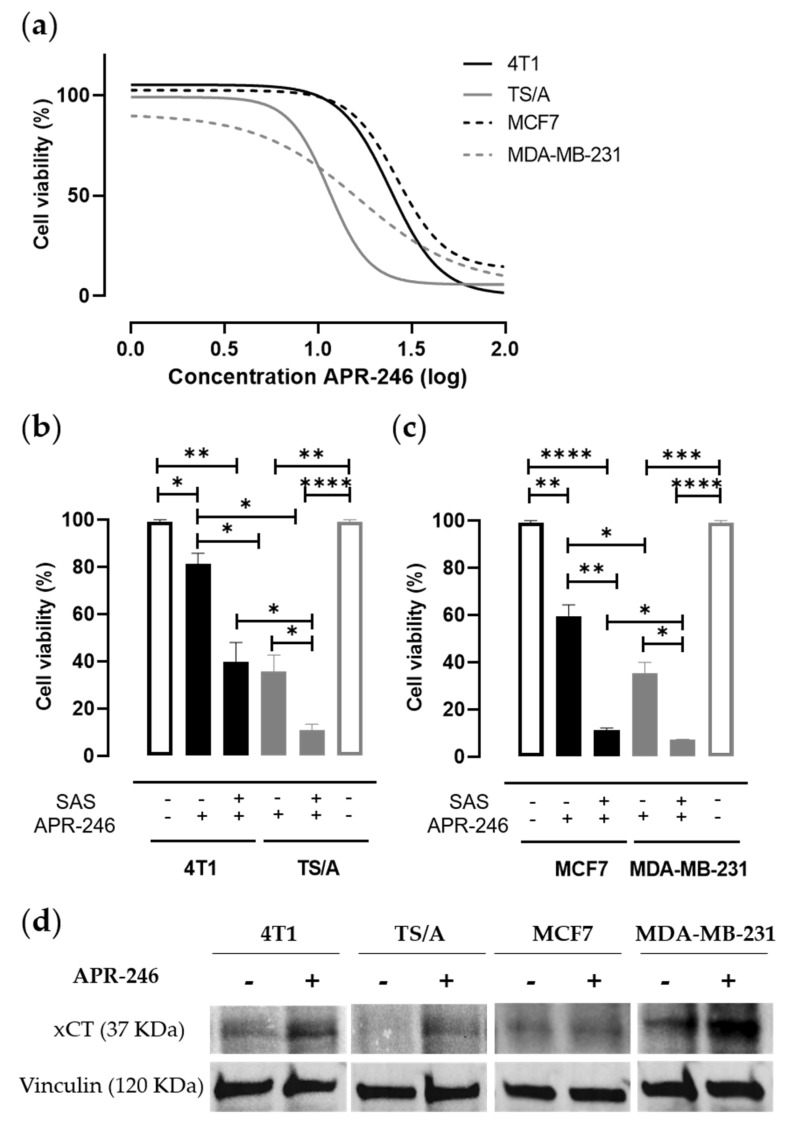
Effect of APR-246 alone or in combination with SAS on mammary tumor cell viability. (**a**) MTT proliferation assay performed on cells cultured for 24 h in the presence of scalar doses of APR-246. The percentage of cell viability measured in treated cells as compared to that measured in untreated cells grown in the presence of medium (no drugs) was calculated. The graph shows log(inhibitor) vs. response-variable slope (four parameters) nonlinear regression of data from three independent experiments, calculated with GraphPad9 software. (**b**,**c**) Histograms represent the percentage (mean value ± mean of the standard error, SEM) of the cell viability following 24 h incubation with 25 µM or 12.5 µM of APR-246 for mut-p53 or p53 wild-type cells, respectively, alone or in combination with 100 µM of SAS. Results are reported in comparison to cells grown in the presence of medium, considered as control (100%). * *p* < 0.05; ** *p* ≤ 0.002; *** *p* = 0.0002; **** *p* < 0.0001; Student *t* test. (**d**) Immunoblot revealing xCT expression in cancer cell line panel treated or not with APR-246 (25 µM for 4T1 and MCF7; 12.5 µM for TS/A and MDA-MB-231) for 16 h. Vinculin was used as loading control protein.

As expected, cell viability was mostly reduced in the mut-p53 cell lines when treated with APR-246 (Figure 1a), showing IC_50_ values in accordance with the literature regarding human cell lines [35] (Table 1). Of note, the combination of APR-246 with SAS was able to reduce IC_50_ value (Supplementary Figure S2) and to significantly decrease cell viability compared to APR-246 treatment alone in all cell lines tested (Figure 1b,c). Similar results have been obtained with the mouse cell lines either by silencing xCT with specific siRNA (Supplementary Figure S3a,b) or by inhibiting xCT with Erastin (Supplementary Figure S3c,d). Overall, these results confirm the action of APR-246 on the mut-p53 but also show its potential action on the cellular redox balance. Consistently, APR-246 can modulate xCT expression by inducing its upregulation in breast cancer cells upon their treatment compared to untreated cells (Figure 1d). This phenomenon could be explained as a compensatory mechanism of cell detoxification since the APR-246-active compound MQ can bind GSH-enhancing ROS levels [25].

Giving this evidence and considering the role of xCT in the redox balance, we evaluated the induction of ROS resulting from APR-246 treatment, alone or in combination with SAS. Even if both SAS and APR-246 alone were able to enhance ROS levels in treated cells, we revealed a trend showing the major enhancement of ROS in the case of the combined treatment (Supplementary Figure S4).

### 3.2. xCT Inhibition Potentiates APR-246 Effectiveness by Inducing Apoptosis of Tumor Cells

**Figure 2:**
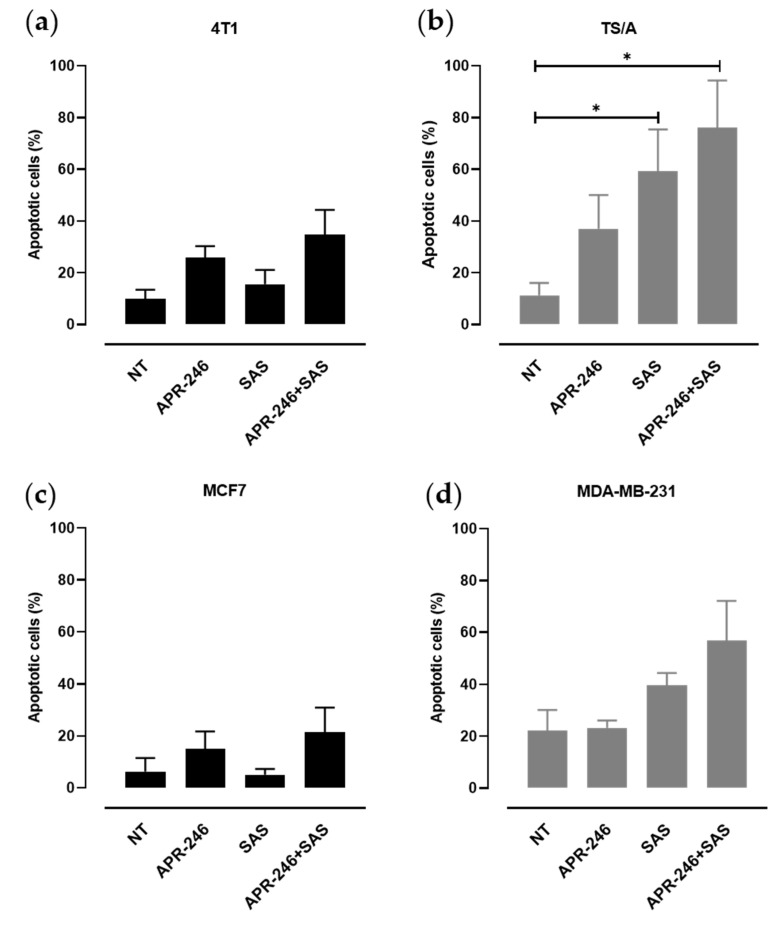
Quantitative analysis of apoptosis induced by APR-246, SAS, or the combinatorial treatment. Values of apoptotic cells for (**a**) 4T1, (**b**) TS/A, (**c**) MCF7, and (**d**) MDA-MB-231 treated or not with APR-246 (5 µM) and/or with SAS (100 µM) for 24 h or not treated (indicated as NT). Data are presented as the mean ± SEM of the percentage of apoptotic cells. * *p* < 0.05; Student’s *t* test.

The expression of xCT, which is induced following cell stress, and the activity of the xc- system represent an adaptive response of tumor cells to re-establish a correct redox balance, thus avoiding cell death. However, p53 is able to negatively regulate xCT protein levels, favoring the apoptotic process [19]. To evaluate the apoptotic response induced by APR-246 in the presence of xCT inhibition in our cell line panel, we exploited the Annexin V/propidium iodide apoptosis assay through cytofluorimetric analysis (Figure 2). SAS treatment induced apoptosis in mut-p53 TS/A and MDA-MB-231 cells, while its effect was limited in p53null or p53 wild-type cells. APR-246 induced a slight increase in apoptosis in all the tested cell lines compared to untreated cells. However, the percentage of cell death further increased upon the combinatorial treatment, even if no statistically significant difference was found between the combination of SAS and APR-246 and the single treatments (Figure 2a–d). These results suggest that mut-p53 sensitizes breast cancer cells to the oxidative stress induced by either SAS or APR-246, and that xCT inhibition, by decreasing the cell antioxidant defenses, potentiates APR-246’s ability to induce apoptosis.

### 3.3. The Combinatorial Treatment with APR-246 and SAS Synergistically Inhibits the Generation of Tumorspheres

**Figure 3:**
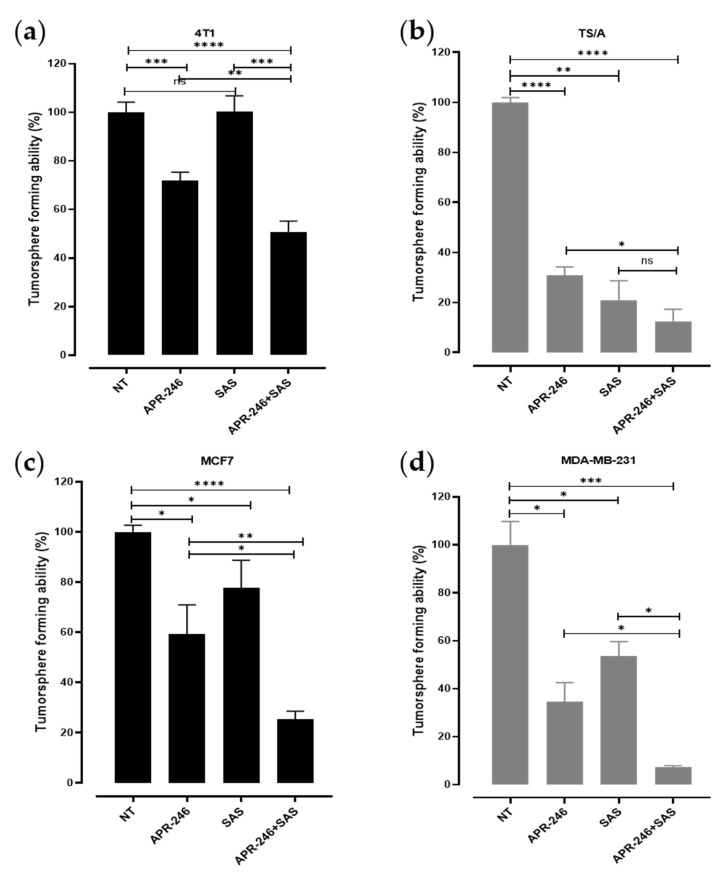
Effect of SAS and APR-246 on tumorsphere generation. Histograms showing the sphere-forming ability of (**a**) 4T1, (**b**) TS/A, (**c**) MCF7, and (**d**) MDA-MB-231 tumor cells treated with APR-246 5 µM, SAS 150 µM, or the combination of the two compounds. Values of treated cells represent the percentage of the tumorsphere-forming ability in comparison to the mean value of the cells cultured in the medium alone, indicated as NT (= not treated), considered as 100%. * *p* ≤ 0.03; ** *p* = 0.001; *** *p* = 0.0001; **** *p* < 0.0001; ns = not significant; Student’s *t* test.

First passage tumorspheres, enriched in CSCs (Supplementary Figure S5), were generated from the various cell lines following the previously established culture conditions [31,32]. Following enzymatic disaggregation, the formation of second passage tumorspheres has been allowed in the presence of SAS or APR-246 alone, revealing a significant decrease in the tumorsphere-forming ability of APR-246-treated cells compared to those cultured with the medium for all the cell lines analyzed. As expected, the effect was greater in mut-p53 TS/A and MDA-MB-231 cells (Figure 3a–d). Treatment with SAS significantly reduced tumorsphere generation in mut-p53 TS/A and MDA-MB-231 cells (Figure 3b,d), while, when used at this concentration, it only induced a mild or no effect in p53 null 4T1 (Figure 3a) and in p53 wild-type MCF-7 (Figure 3d) cells, confirming the higher sensitivity of cells harboring the p53 mutation to redox balance alteration. The combinatorial treatment with SAS and APR-246 showed a greater effect compared to SAS or APR-246 alone, significantly impairing the self-renewal ability of CSCs from all the cell lines analyzed independently of their p53 status.

### 3.4. xCT Immune Targeting Coupled with APR-246 Treatment Hampers Tumor Growth and Lung Metastasis Formation in Preclinical Models of Breast Cancer

**Figure 4:**
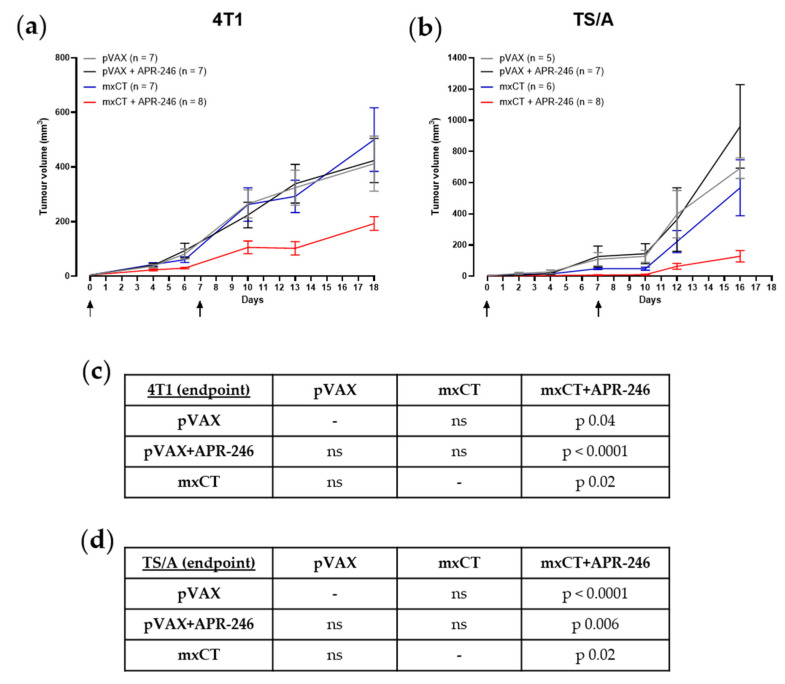
Therapeutic effect of APR-246 and anti-xCT immunization on mouse mammary tumors. Average tumor growth rate (expressed as tumor volume in mm^3^; mean ± SEM for each group) of s.c. (**a**) 4T1- and (**b**) TS/A-induced tumors in BALB/c mice electroporated with pVAX control plasmid alone (grey lines) or in combination with APR-246 treatment (100 mg/Kg i.p. daily, starting when the tumor was palpable; black lines); immunized with mxCT plasmid alone (blue lines) or in combination with APR-246 treatment (red lines); “n” indicates the number of animals per group. The black arrows indicate the immunization timing. (**c**,**d**) Tables reporting the statistical differences between the experimental groups of mice challenged with 4T1 and TS/A, respectively, at the endpoint, analyzed with the Student’s *t* test (ns = not significant).

The therapeutic potential of the treatment with APR-246 was tested in vivo, alone, or in combination with anti-xCT DNA electrovaccination. Mammary tumors were induced in BALB/c mice using a subcutaneous injection (s.c.) of 4T1 or TS/A cells. Starting when the tumor was palpable (2 mm mean diameter), APR-246 was administered daily by intraperitoneal injection in combination with the electroporation of the pVAX empty control vector or of the mxCT plasmid [4] twice with a week interval. Other groups of tumor-bearing mice were immunized against xCT or were electroporated with pVAX without the administration of APR-246, representing the experimental controls. While neither APR-246 administration nor mxCT-based vaccination alone were able to induce a slow-down in the growth of both 4T1 and TS/A tumors compared to the pVAX control group (Figure 4a,b; upper panels), tumor-bearing mice that underwent xCT immunization and were treated with APR-246 displayed a significant reduction in tumor growth (Figure 4a,b; upper panels; lower panels).

**Figure 5:**
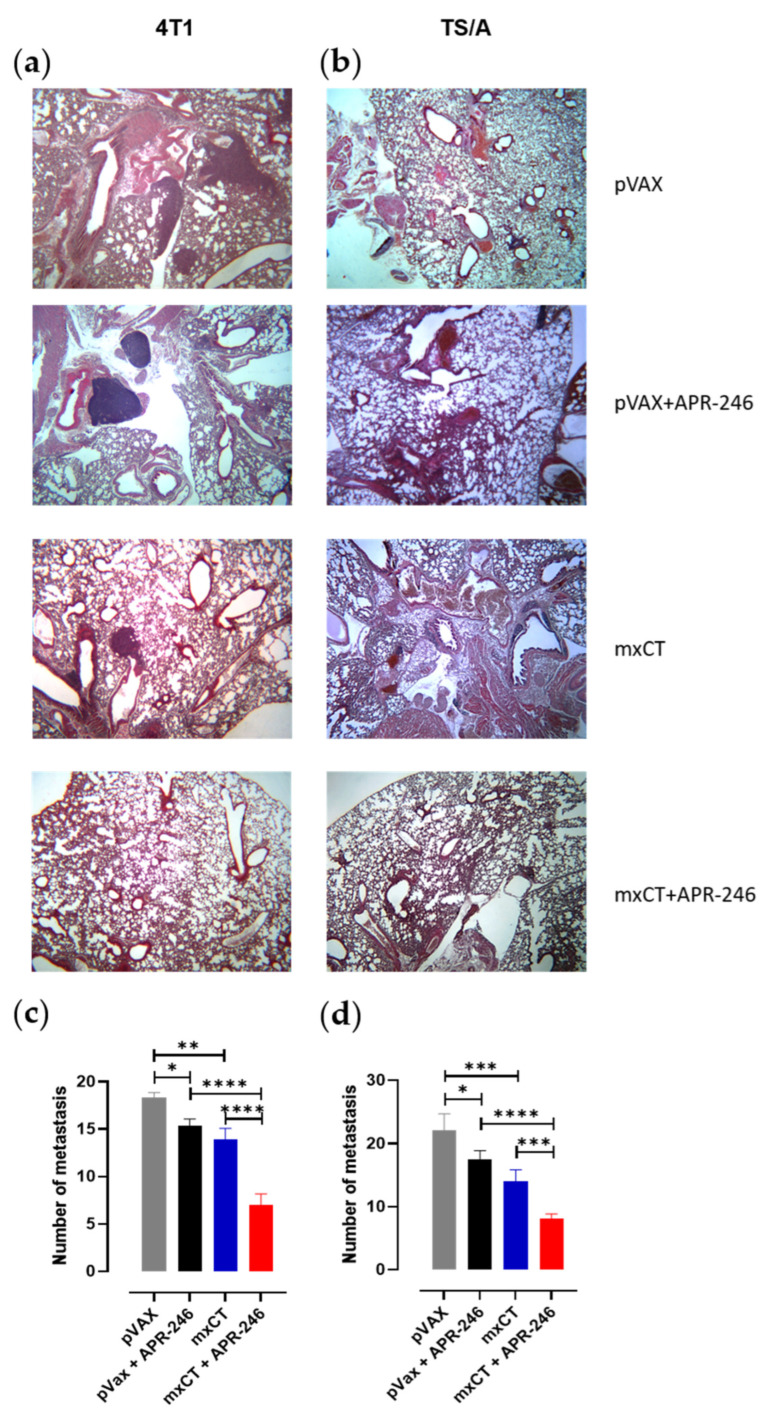
Effect of APR-246 and anti-xCT immunization on lung metastases formation. Histology of spontaneous lung metastases in BALB/c mice challenged s.c. with (**a**) 4T1 or (**b**) TS/A and treated with APR-246 in combination with the electroporation of pVAX control plasmid or of mxCT plasmid. The lungs were collected at the experimental endpoint and formalin-fixed, paraffin-embedded sections were processed for H&E staining; representative pictures are shown (Olympus BX41 microscope; 2.5× magnification). Lung metastases of (**c**) 4T1- or (**d**) TS/A-bearing mice that underwent the different treatments have been quantified; histograms represent the mean value ± SEM of the number of metastases manually counted with a Nikon SMZ1000 stereomicroscope in two slices per sample obtained from the different groups of mice. * *p* ≤ 0.04; ** *p* = 0.004; *** *p* = 0.0002; **** *p* < 0.0001; Student’s *t* test.

Moreover, the number of spontaneous lung metastases was lower in the tumor-bearing mice that received the combination treatment compared to those that received the single treatments, both in 4T1 and TS/A experimental groups (Figure 5a–d), even if xCT immunization alone was able to reduce lung metastatization compared to pVAX, in line with our previous results [4].

### 3.5. DNA Vaccination against xCT Induces an Antibody Response Driving ADCC

**Figure 6:**
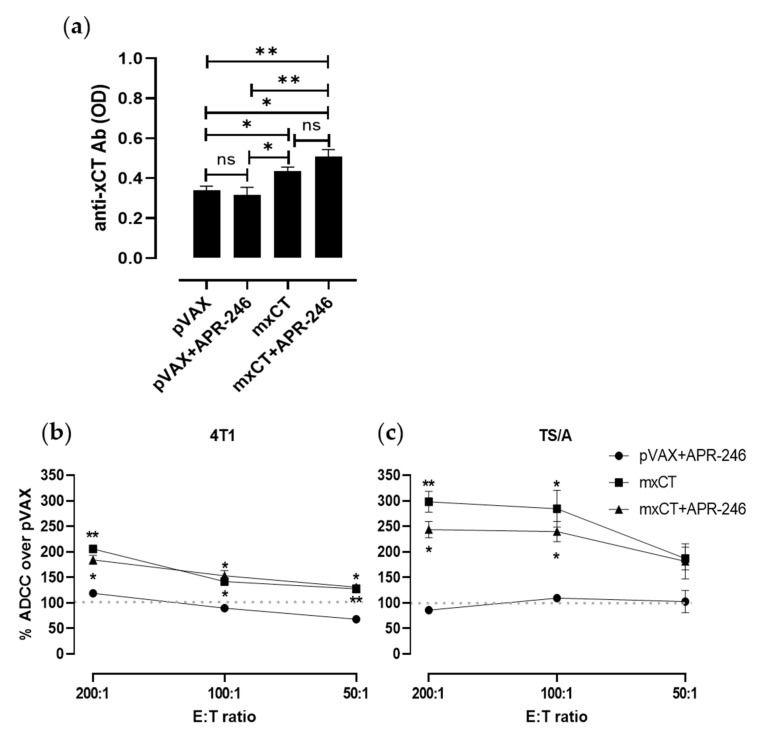
Immune response upon anti-xCT immunization in combination with APR-246: (**a**) Detection of vaccination-induced anti-xCT antibodies by ELISA in sera collected from the indicated groups of mice. Results are shown as mean ± SEM of optical density (OD) values obtained in triplicate. In vitro ADCC assays performed in the presence of serum from mice immunized with mxCT alone or in combination with APR-246 using (**b**) 4T1 and (**c**) TS/A tumor cells as targets (T) and splenocytes from syngeneic BALB/c mice as effectors (E) (E:T ratio = 200:1; 100:1; 50:1). Results are expressed as referred to values of lysis recorded with pVAX control mice serum (dotted lines). * *p* ≤ 0.04; ** *p* ≤ 0.006. Student’s *t* test.

To evaluate the antibody response induced by anti-xCT vaccination, sera collected from immunized mice or from pVAX electroporated control mice were tested by a xCT-specific ELISA. Anti-xCT IgG were detected in the sera of mice vaccinated with mxCT plasmid alone and in combination with APR-246 in a significantly higher amount compared to the control mice (Figure 6a). This result demonstrates that APR-246 does not impair the induction of a proper immune response. The antibodies induced by anti-xCT vaccination, alone or in combination with APR-246, can mediate an effective ADCC response against both 4T1 (Figure 6b) and TS/A (Figure 6c) tumor cells. This finding demonstrates that APR-246-based treatment does not influence the ADCC response and that anti-xCT immune-targeting can potentiate the effect of APR-246.

## 4. Discussion

The urgency to find new valid therapies to face breast cancer, especially the more aggressive histotypes for which therapeutic approaches leading to a significant extension of the overall survival time and to the absence of recurrences are still missing, is spurring the scientific community to look for more personalized and sophisticated approaches.

Here, we evaluated in vitro and in vivo the effectiveness of a new combinatorial therapeutic strategy, based on the inhibition of xCT together with APR-246 treatment, with the aim of restoring the wild-type form of p53 in different breast cancer cell lines. The results we obtained show that the combinatorial strategy led to a reduction in tumor cell viability, the induction of apoptosis, and the impairment of tumorsphere maintenance to a greater extent than the single treatments. Additionally, we achieved relevant in vivo findings in preclinical models, showing that xCT immunetargeting in combination with APR-246 treatment hampers breast tumor progression and metastatization.

As we previously demonstrated, xCT is overexpressed in breast CSCs and participates in their self-renewal and drug resistance processes [4]. The relevant role of xCT as a critical molecule in carcinogenesis, tumor invasion, and patients’ prognosis has been confirmed also in other cancer types, such as nonsmall cell lung cancer [36], hepatocellular carcinoma [37,38], colorectal cancer [39], and oral [40] and liver carcinoma [41]. Despite all this evidence, the mechanisms underlying the role of xCT in tumorigenesis largely remains unknown. Certainly, xCT actively participates in the GSH biosynthesis process, which contributes to metabolic reprogramming and chemoresistance, mechanisms of protection exploited by cancer cells to avoid oxidative stress and ferroptosis, a form of nonapoptotic cell death implying the accumulation of lipid peroxides and ROS through iron catalysis [42]. Indeed, xCT constitutive activation has been demonstrated to suppress ferroptosis [42,43], whereas xCT inhibition brought on using its inhibitors can induce ferroptosis in cancer cells. In this scenario, xCT targeting may represent a worthwhile contribution in cancer therapy, as demonstrated by several studies on xCT inhibitors [44,45,46,47,48,49]. Indeed, xCT has attracted the attention of pharmaceutical companies that are trying to develop new inhibitors for cancer treatment [50], since the currently available xCT inhibitory molecules, including SAS, Sorafenib, Erastin, and IKE, present some limitations regarding their use in vivo, offering poor pharmacokinetics and bioavailability. Therefore, currently, their use in patients is still limited.

xCT is only expressed on a few normal cells, such as astrocytes and some myeloid cells [5]. It is therefore a good target for vaccination, which represents a promising approach for cancer treatment, being a cost-effective strategy [3]. xCT has revealed as a valuable target for immunotherapy, as demonstrated by our previous works describing the use of anti-xCT DNA-, viral vector based-, or virus-like particle-based vaccines as effective in inducing an immune response able to hamper xCT function, causing ROS accumulation in breast CSCs [4,5,11,12,13]. Indeed, even if anti-xCT immunization alone has been demonstrated to be unable to induce cancer remission, it can act against the dissemination process that leads to metastatization. Importantly, no adverse events were observed in xCT-vaccinated mice [4,12], and no detrimental organ alterations nor alterations or immune infiltration in the central nervous system were observed [11], suggesting that anti-xCT vaccines are safe.

These findings demonstrate the potential of xCT as a cancer immunotherapeutic target that may be used in combination with conventional or new therapeutic strategies. Of note, we recently demonstrated that the immunetargeting of xCT is able to potentiate a viral vector-based vaccine against Her2 in constraining Her2^+^ breast tumor growth as well as in decreasing CSC frequency and metastatic events in preclinical mouse models [14]. However, since Her2^+^ breast cancer represents only 20% of breast cancer histotypes, it remains crucial to explore new combinatorial strategies tailored to other breast cancer types, such as those missing the expression of easily targetable antigens.

Considering that cancer treatment requires the simultaneous modulation of different pathways and biological functions playing pivotal roles in maintaining cell homeostasis, redox balance, and regulating cell death, xCT targeting, together with the reactivation of p53, can be the right track to pursue. Indeed, the *TP53* tumor suppressor gene is prone to a loss of function and to missense mutations in human cancers. In particular, these mutations occur in 30% of all breast cancers, making it the most mutated gene in this disease [51].

**Figure 7:**
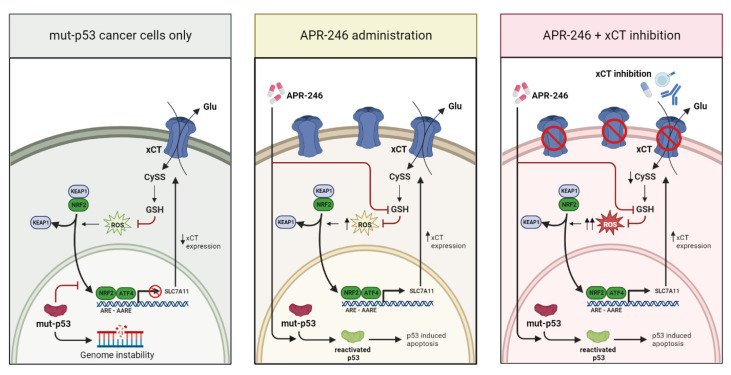
Schematic representation of xCT and mut-p53 interplay in presence or absence of APR-246 treatment. Left panel: p53-induced modification in mut-p53 cancer cells; central panel: alterations induced by APR-246 administration include the restoring of xCT expression and the inhibition of GSH; right panel: cellular alteration induced by the combination of APR-246 administration and xCT inhibition. Long black arrows: induction; red lines: inhibition; short black arrows up: xCT upregulation; short black arrows down: xCT downregulation; inhibited circle: loss of xCT expression.

Of note, compelling insights have emerged into the link between the mechanisms regulating xCT gene expression and that of p53 and mut-p53, as reviewed in [15]. Specifically, xCT expression can be repressed by wild type p53, regulating ferroptosis [16]. However, transcription factors involved in the oxidative stress response, such as NRF2 and activating transcription factor 4 (ATF4), can counterbalance xCT expression towards upregulation. On the other hand, a mechanism of the regulation of *SLC7A11* by mut-p53 has also been elucidated, showing that mut-p53 is able to bind NRF2, preventing xCT transcription [25] (Figure 7, let panel). To enrich this framework, it has been convincingly demonstrated that low expression levels of xCT are strongly predictive for sensitivity to APR-246 [23], the first-in class reactivator of mut-p53 that we used in this study. The ability of APR-246 to react with thiol-containing molecules, such as cysteine residues in the core domain of mut-p53 through its active intermediate MQ, leads to the conformational change of the mutated protein, re-enabling the proapoptotic program of p53 [52]. In fact, the largest part of clinical trials involving APR-246 concerns malignancies carrying *TP53* mutations, with a focus on hematologic tumors [21,53]. Thus, treatment with APR-246 may impair mut-p53-dependent NRF2 inhibition of xCT, increasing its expression (Figure 7, central panel), as shown by [25] using esophageal cancer cells and as confirmed here by our data on breast cancer. However, the antitumor activity of APR-246 seems to lean also on the perturbation of the antioxidant pathways due to GSH targeting and depletion [25,26], which triggers ferroptosis [23]. Indeed, we observed xCT upregulation in cells treated with APR-246 even in the absence of mut-p53. Similar results have been demonstrated in acute myeloid leukemia cells harboring wild type p53 [54] and in breast cancer cell lines at the transcriptional level [55]. These findings suggest a p53-independent action of APR-246 in regulating xCT expression (Figure 7, central panel). The upregulation of genes involved in redox balance maintenance, including *SLC7A11*, can be interpreted as a cellular stratagem to restore GSH homeostasis upon treatments eroding the GSH pool and leading to ROS accumulation. Another explanation could be the rollout of compensatory mechanisms favoring xCT transcription, such as the induction of AT-rich interacting domain-containing protein 1A (ARID1A), which forms a complex on the *SLC7A11* promoter with NRF2 and other SWI/SNF complex subunits, regulating xCT transcription [56]. Therefore, an APR-246-mediated increase in xCT sensitizes tumor cells to xCT targeting.

Since it has been shown that treatment with APR-246 consumes GSH reserves and causes an increase in ROS [25], the simultaneous blockade of xCT function, not allowing cancer cells to counterbalance this oxidative stress, exerts a synergistic effect (Figure 7, right panel). Of note, xCT immunetargeting was effective in synergizing with APR-246 in vivo in the mouse models of breast cancer, demonstrating that it may be a successful alternative to xCT inhibitors in vivo. In particular, vaccination induces anti-xCT-specific antibodies that can target cancer cells by at least two distinct mechanisms, i.e., the direct effect on cancer cells mediated by their ability to inhibit xCT [4,11,12,13] and immune-mediated effects such as the induction of ADCC. This is particularly important for the elimination of CSCs, which downregulate MHC class I and are thus able to escape from T cell killing [57]. The combined treatment is effective not only in mut-p53 TS/A tumor-bearing mice, but also in the p53-null 4T1 model, in line with the previous observations that APR-246 is able to induce cytotoxic effects even when administered to p53-null or p53-knockdown cells, thanks to its ability to bind GSH and thus alter cell redox balance [58].

It is interesting to note that APR-26 did not impair the immune response induced by xCT vaccination. This contradicts what was observed by Zhang et al. [59], who used APR-246 in combination with dendritic cell-based vaccination against p53 in a methylcholanthrene-induced primary murine tumor model without observing a significant effect of the combined therapy in reducing tumor-free survival in vivo. The researchers ascribed this failure to the inhibition of immune cells induced by APR-246, which was not observed in our experimental setting. This discrepancy may be due to the different administration schedules, since Zhang et al. administered APR-246 for a longer time (60 days vs. 18 days) and through a different route (oral vs. i.p.) [59], or to a different sensitivity of the cancer models used.

In conclusion, our findings demonstrate that xCT vaccination synergized with drugs is able to restore the function of mut-p53 to limit breast cancer progression. This approach could be further combined with therapies such as immune checkpoint inhibitors or chemotherapy to induce greater anticancer immune responses, providing new options in the management of breast cancer patients.

## Figures and Tables

**Table 1:** APR-246 IC_50_ values.

Cell Line	p53 *Status*	APR-246 *	APR-246 + SAS *
4T1	*Null*	24.4	10.8
TS/A	mutated (R270H)	11.6	4.2
MCF7	wild type	26.6	16.6
MDA-MB-231	mutated (R280K)	9.2	4.5

* APR-246 IC_50_ (µM) based on the indicated treatment.

## Data Availability

The data presented in this study are available in the Article and Supplementary materials.

## Supplementary Materials

The following supporting information can be downloaded at: https://www.mdpi.com/article/10.3390/biomedicines10112843/s1, Figure S1: Assessment of xCT expression in breast cancer cell line panel; Figure S2: MTT assays to determine the IC_50_ value of APR-246 and its effect on cell viability; Figure S3: Effect of APR-246 in combination with xCT-depleted mouse mammary tumor cells; Figure S4: Cytofluorimetric analysis of intracellular ROS in treated cells; Figure S5: Tumorspheres are enriched in cancer stem cells.
