# Supplementary material for: Immunotherapy against the Cystine/Glutamate Antiporter xCT Improves the Efficacy of APR-246 in Preclinical Breast Cancer Models
Source: Biomedicines. 2022 Nov 8;10(11):2843. doi: 10.3390/biomedicines10112843 (PMC9688020; doi:10.3390/biomedicines10112843)
Supplement: Supplementary file 1 [file biomedicines-10-02843-s001.zip › Supplementary file.pdf]

Supplementary figures: Immunotherapy against the cystine/glutamate antiporter xCT improves the efficacy of APR-246 in preclinical breast cancer models.

Giuseppina Barutello, Antonino Di Lorenzo, Alessandro Gasparetto, Chiara Galiazzi, Elisabetta Bolli, Laura Conti and Federica Cavallo

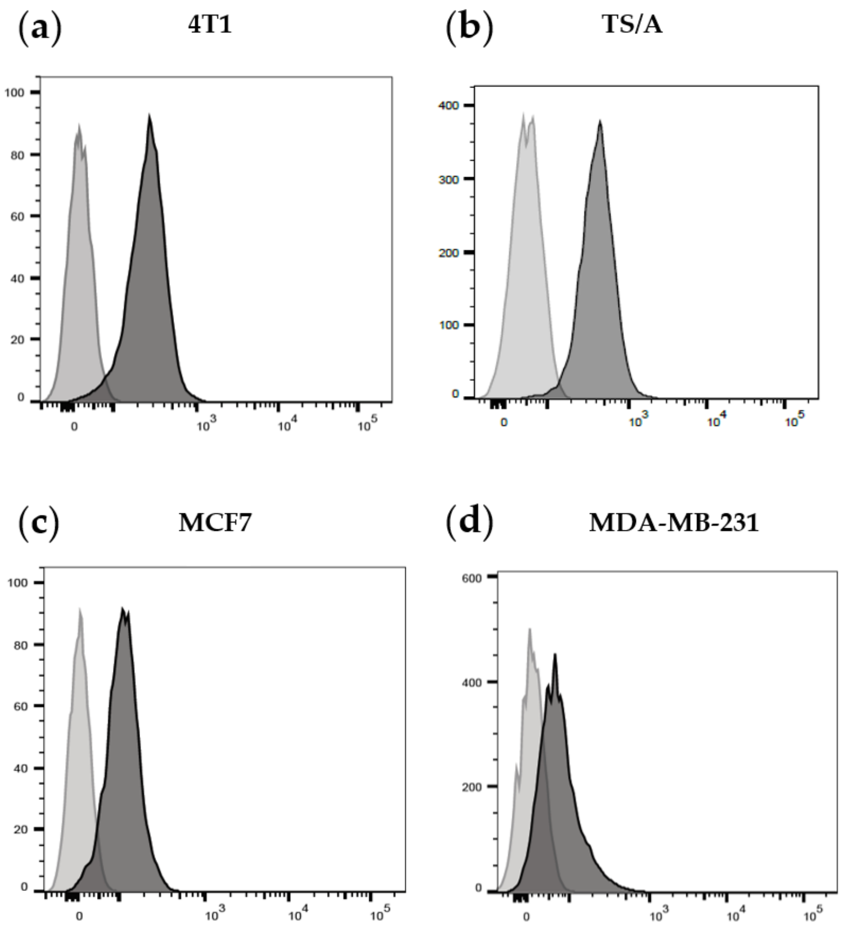

Figure S1. **Assessment of xCT expression in breast cancer cell line panel.** Representative FACS histograms of xCT (dark grey) in comparison to the control antibody control (light grey) staining on (**a**) 4T1, (**b**) TS/A, (**c**) MCF7, and (**d**) MDA-MB-231 cells.

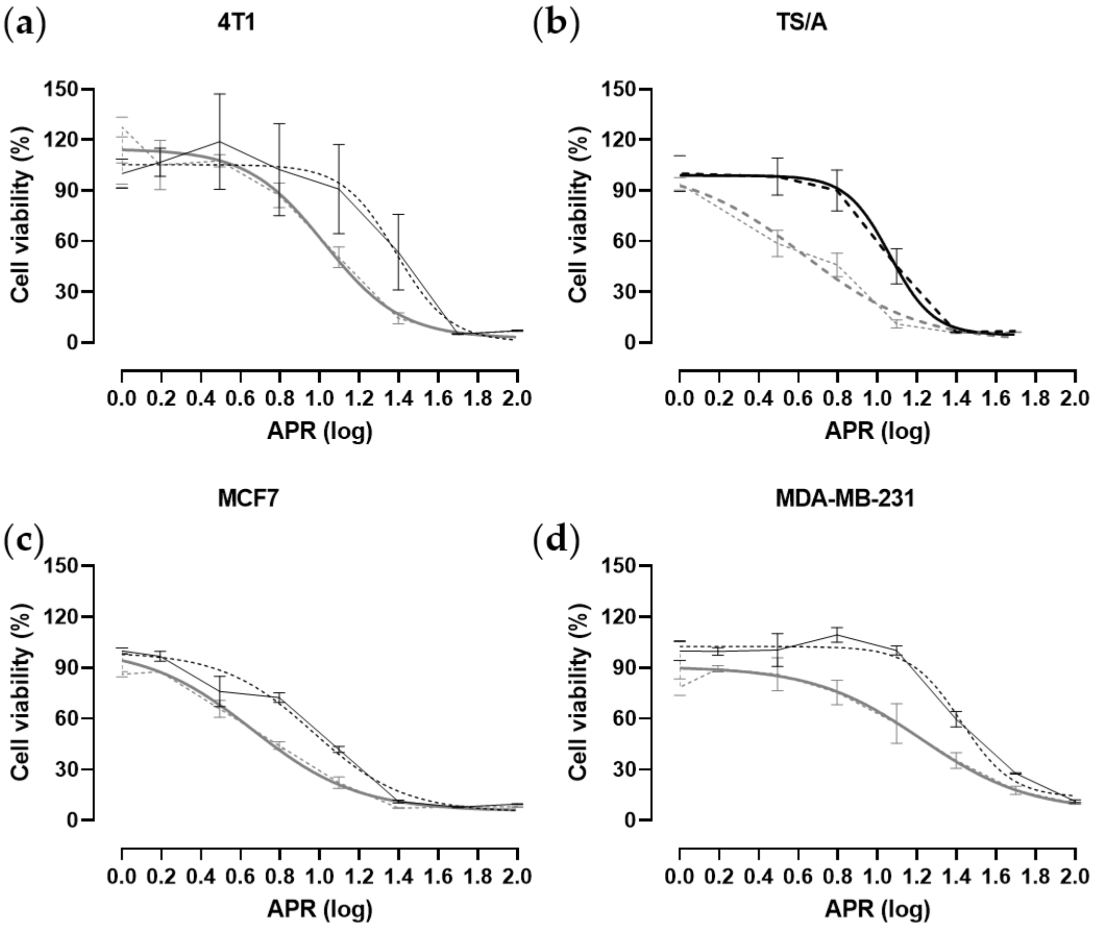

Figure S2. MTT assays to determine the IC50 value of APR-246 and its effect on cell viability. The results are expressed as the percentage of cell viability of cells following 24 hours incubation with APR-246 alone (black lines) or in combination with SAS (grey lines). The graphs show log(inhibitor) vs. response - variable slope (four parameters) non-linear regression of data from three independent experiments, calculated with GrapPad9 software.

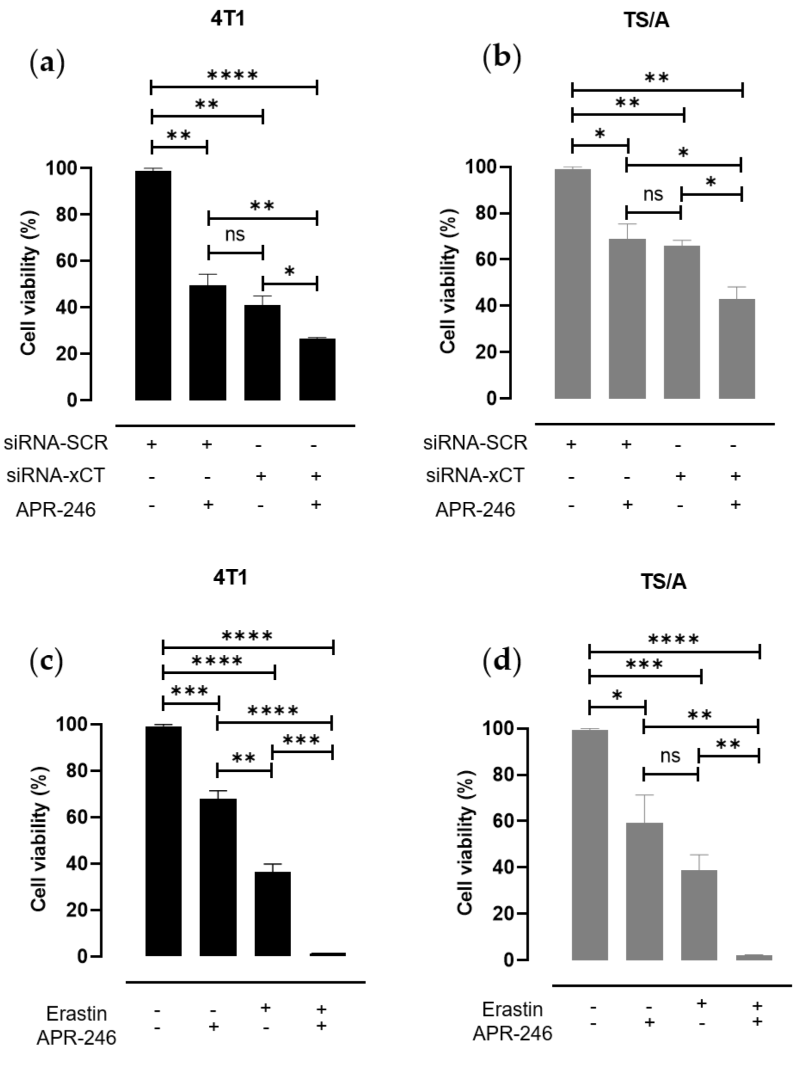

Figure S3. Effect of APR-246 in combination with xCT depleted mouse mammary tumor cells. Histograms represent the percentage (mean value ± SEM) of the cell viability of (**a**) 4T1 and (**b**) TS/A previously treated with specific mouse xCT siRNA mix (siRNA-xCT), following 24 hours incubation with 25 μM or 12.5 μM of APR-246 respectively, and of (**c**) 4T1 and (**d**) TS/A following 24 hours incubation with APR-246 alone, or in combination with 0.2 μM of Erastin. Results are reported in comparison to cells grown in the presence of medium added with the control siRNA (siRNA-SCR) (**a, b**) or medium (**c, d**), considered as control (100%). *p = 0.02; **p ≤ 0.009; ***p = 0.0008; ****p < 0.0001; ns = not significant; Student t test.

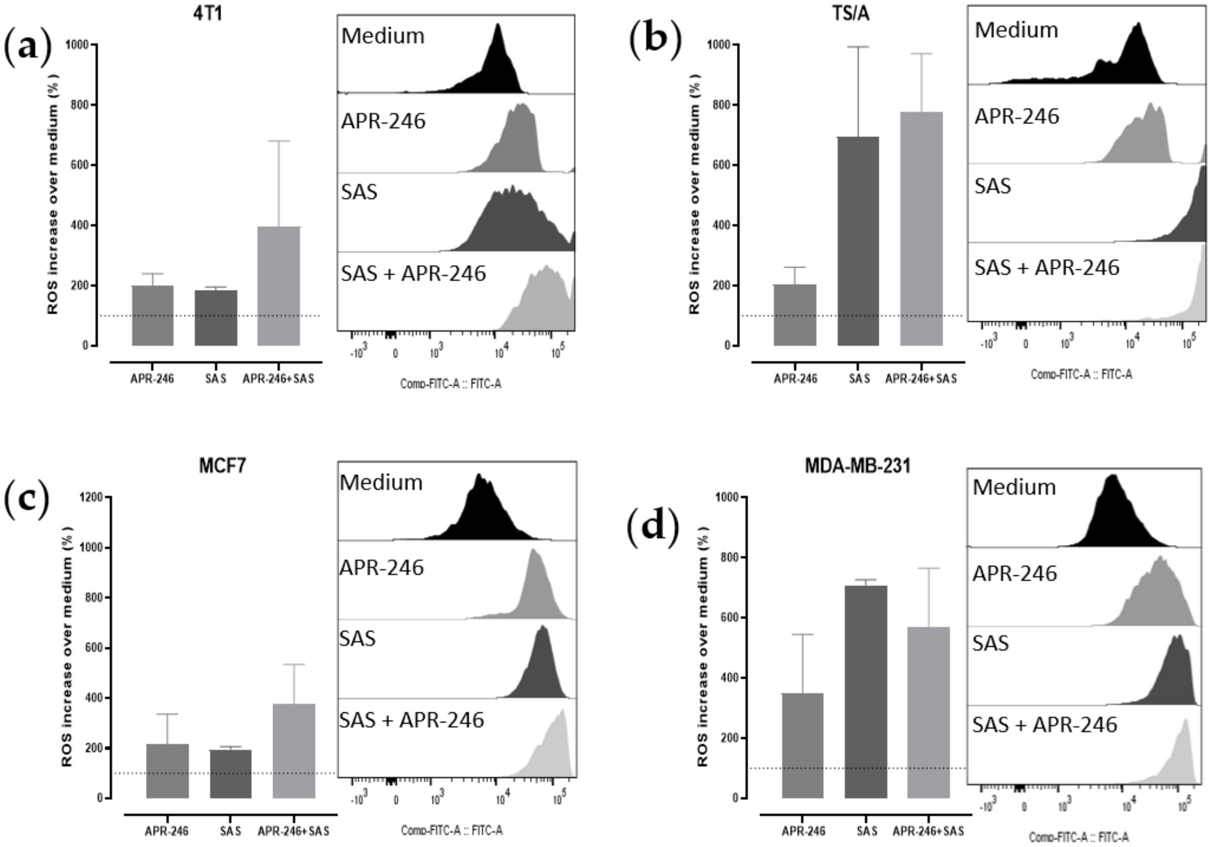

Figure S4. Cytofluorimetric analysis of intracellular ROS in treated cells. FACS analysis of intracellular ROS in (**a**) 4T1, (**b**) TS/A, (**c**) MCF7, and (**d**) MDA-MB-231 cells treated with APR-246 and/or SAS, reported as percentage of increase of the DCF MFI in treated cells as compared to cells cultured in medium (means ± SEM from 3 independent experiments) and asrepresentative FACS histograms.

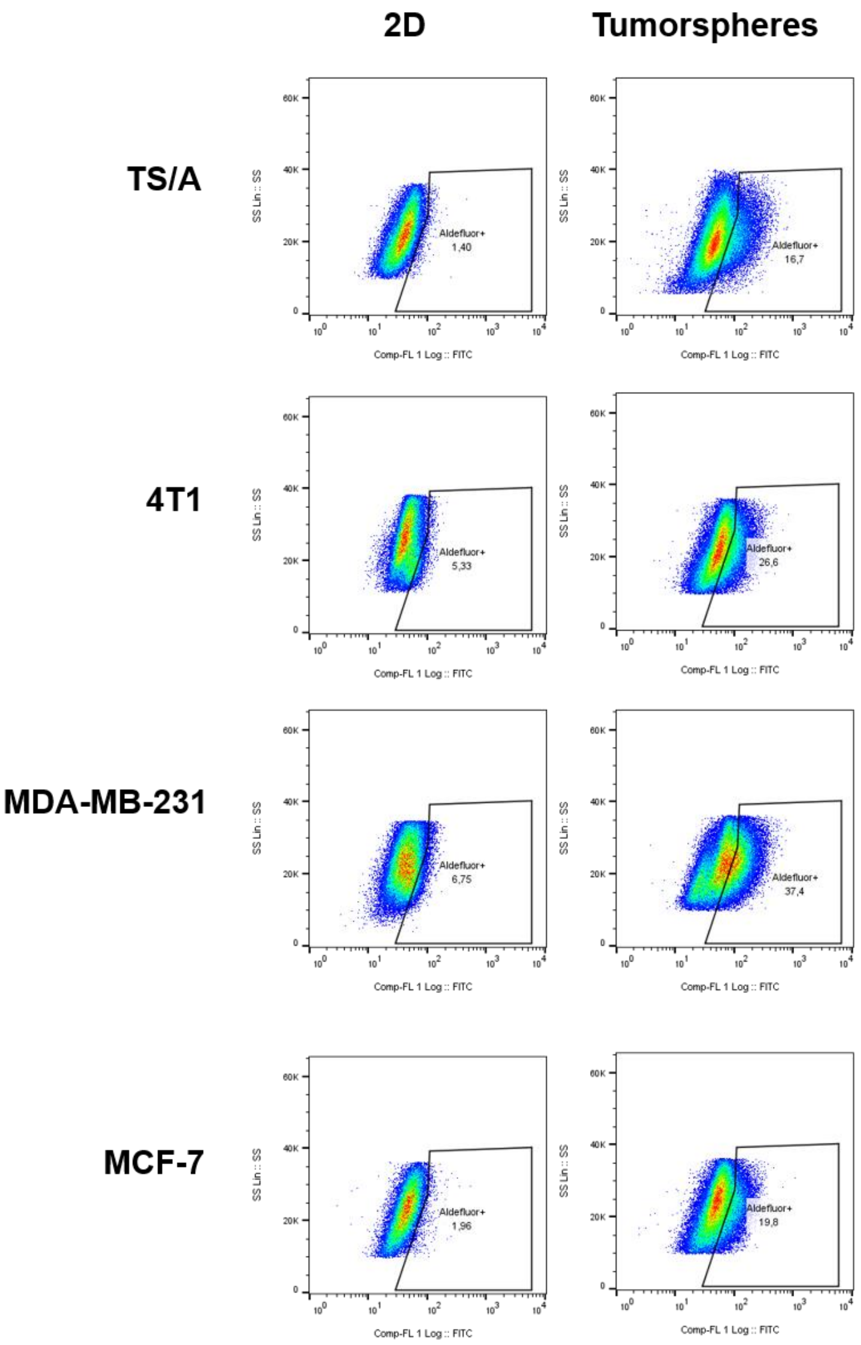

Figure S5. Tumorspheres are enriched in cancer stem cells. FACS analysis of aldehyde dehydrogenase 1 activity, measured using the Aldefluor kit (Stem Cell Technologiesis), in TS/A, 4T1, MDA-MB-231 and MCF-7 cells cultured in standard 2D conditions or as tumorspheres. Representative density plots are shown. The gates were set based on the corresponding control samples, and the percentage of Aldefluor$^+$ cells is reported.
